# An arginase1- and PD-L1-derived peptide-based vaccine for myeloproliferative neoplasms: A first-in-man clinical trial

**DOI:** 10.3389/fimmu.2023.1117466

**Published:** 2023-02-23

**Authors:** Jacob Handlos Grauslund, Morten Orebo Holmström, Evelina Martinenaite, Thomas Landkildehus Lisle, Hannah Jorinde Glöckner, Daniel El Fassi, Uffe Klausen, Rasmus E. J. Mortensen, Nicolai Jørgensen, Lasse Kjær, Vibe Skov, Inge Marie Svane, Hans Carl Hasselbalch, Mads Hald Andersen

**Affiliations:** ^1^ National Center for Cancer Immune Therapy (CCIT-DK), Department of Oncology, Copenhagen University Hospital, Herlev, Denmark; ^2^ Institute for Immunology and Microbiology, University of Copenhagen, Copenhagen, Denmark; ^3^ Research and Development, IO Biotech ApS, Copenhagen, Denmark; ^4^ Department of Hematology, Copenhagen University Hospital, Rigshospitalet, Copenhagen, Denmark; ^5^ Department of Hematology, Zealand University Hospital, Roskilde, Denmark

**Keywords:** myeloproliferative neoplasms, cancer immune therapy, arginase-1, PD-L1, immune modulatory vaccines

## Abstract

**Introduction:**

Arginase-1 (ARG1) and Programed death ligand-1 (PD-L1) play a vital role in immunosuppression in myeloproliferative neoplasms (MPNs) and directly inhibit T-cell activation and proliferation. We previously identified spontaneous T-cell responses towards PD-L1 and ARG1 derived peptide epitopes in patients with MPNs. In the present First-in-Man study we tested dual vaccinations of ARG1- derived and PD-L1-derived peptides, combined with Montanide ISA-51 as adjuvant, in patients with Janus Kinase 2 (JAK2) V617F-mutated MPN.

**Methods:**

Safety and efficacy of vaccination with ARG1- derived and PD-L1-derived peptides with montanide as an adjuvant was tested in 9 patients with MPN The primary end point was safety and toxicity evaluation. The secondary end point was assessment of the immune response to the vaccination epitope (www.clinicaltrials.gov identifier NCT04051307).

**Results:**

The study included 9 patients with *JAK2*-mutant MPN of which 8 received all 24 planned vaccines within a 9-month treatment period. Patients reported only grade 1 and 2 vaccine related adverse events. No alterations in peripheral blood counts were identified, and serial measurements of the JAK2V617F allelic burden showed that none of the patients achieved a molecular response during the treatment period. The vaccines induced strong immune responses against both ARG1 and PD-L1- derived epitopes in the peripheral blood of all patients, and vaccine-specific skin-infiltrating lymphocytes from 5/6 patients could be expanded in vitro after a delayed-type hypersensitivity test. In two patients we also detected both ARG1- and PD-L1-specific T cells in bone marrow samples at the end of trial. Intracellular cytokine staining revealed IFNγ and TNFγ producing CD4^+^- and CD8^+^- T cells specific against both vaccine epitopes. Throughout the study, the peripheral CD8/CD4 ratio increased significantly, and the CD8^+^ TEMRA subpopulation was enlarged. We also identified a significant decrease in PD-L1 mRNA expression in CD14^+^ myeloid cells in the peripheral blood in all treated patients and a decrease in ARG1 mRNA expression in bone marrow of 6 out of 7 evaluated patients.

**Conclusion:**

Overall, the ARG1- and PD-L1-derived vaccines were safe and tolerable and induced strong T-cell responses in all patients. These results warrant further studies of the vaccine in other settings or in combination with additional immune-activating treatments.

## Introduction

Philadelphia chromosome-negative myeloproliferative neoplasms (MPNs) include essential thrombocythemia (ET), polycythemia vera (PV), and primary myelofibrosis. These three diseases almost exclusively exhibit certain driver mutations: the *JAK2V617F* point mutation, the calreticulin gene (*CALR*) frameshift mutations, or the thrombopoietin receptor gene (*MPL*) mutation. The treatment of MPN is aimed at reducing the risk of thromboembolic episodes through lifestyle intervention, low-dose aspirin, and cytoreductive therapies such as hydroxyurea (HU) and pegylated interferon alpha (IFN-α). Patients with PV and ET have a 5-10% risk of developing acute myeloid leukemia within 20 years (1). Currently, the only available curative treatment modality is allogeneic hematopoietic stem cell transplantation (alloHSC). This treatment is, however, associated with high mortality and is thus only used in patients with high or intermediate risk myelofibrosis (2).

During the last decade cancer immune therapy based on targeting immunosuppressive mechanisms has shown great potential in treatment of solid tumors (3) and several of the key known immunosuppressive pathways such as programmed death ligand-1 (PD-L1) and arginase1 (ARG1) have also been identified in MPN (4–6). Thus, cancer immune therapies targeting these suppressive mechanisms could be a potential new treatment option for MPN.

PD-L1 is upregulated on either tumor cells or tumor-infiltrating cells in many cancers and is known to diminish T-cell activation through its interaction with programmed death receptor 1 (PD-1) on T cells (7). Antibodies that block the PD-1:PD-L1 axis have been effective in different types of cancers, including hematologic malignancies (7–9), but these drugs can have severe side effects (10). Prestipino et al. showed that the *JAK2*V617F mutation in MPNs upregulates PD-L1 by activation of STAT3 and STAT5, transcription factors for the *CD274* gene, and thereby mediates immune escape in MPN (5). Other studies have shown that PD-L1 expression is increased in patients with MPN compared to healthy controls, regardless of the driver mutation (11–13). Arginase-1 (ARG1), on the other hand, is often expressed by regulatory immune cells such myeloid-derived suppressor cells (MDSC) and exerts its suppressive function reducing the availability of L-arginine in the tumor microenvironment and thus causing the downregulation of the CD3ζ chain, and inhibiting T-cell proliferation (14, 15). It has been shown that patients with MPN have higher levels of MDSCs and generally higher ARG1 mRNA expression levels in peripheral blood compared to healthy controls (6).

T cells that specifically target epitopes derived from immune suppressive protein expressed by immune-suppressive cells are defined as anti-Tregs (16). Immunogenic epitopes from multiple immunosuppressive proteins such as indoleamine 2,3-dioxygenase (IDO) (17), ARG1, and PD-L1 have been identified and characterized (18, 19). Anti-Tregs appear to be important for immune homeostasis, due to their ability to directly react against regulatory immune cells (16) by suppressing their inhibitory function and promoting a more pro-inflammatory microenvironment (18, 20). Accordingly, we reasoned that, by activating anti-Tregs specific for PD-L1 and ARG1 derived epitopes through peptide vaccination, it may be an effective strategy to reinstate immune homeostasis in patients with MPN by immunomodulation of the tumor microenvironment and promotion of the tumor-specific T cell responses.

In 2013, we identified immunogenic peptide epitopes in PD-L1 (21) and spontaneous T-cell responses against these epitopes were identified in peripheral blood mononuclear cells (PBMC) from both healthy controls and patients with cancer (21). These specific T cells were able to kill melanoma cell lines as well as dendritic cells, in a PD-L1-dependent manner (21). and enhanced virus- and cancer-specific T-cell responses *in vitro* (18, 22). Similarly, we identified a 50-amino acid (50-aa) hotspot region of immunogenic epitopes in the ARG1 protein (aa 161-210) (19). Exploration of this hotspot region identified a 38-aa peptide, ‘ArgLong2’, that activated frequent, strong, and spontaneous ARG1-specific T-cell responses in PBMC samples from heathy donors as well as cancer patients (20, 23). We frequently observed spontaneous responses against ARG1 and PD-L1-derived peptides in T cells from patients with MPN (12, 24).

Among the immunogenic peptides identified in PD-L1, PD-L1Long1 was tested previously in four clinical trials. One trial was performed in ten patients with multiple myeloma after high dose chemotherapy and autologous hematopoietic stem cell transplantation. Of the 10 patients vaccinated, three showed clinical improvement (25). The second trial included patients with basal cell carcinoma, where a PD-L1Long1 vaccine, in combination with Montanide ISA-51, showed a profound effect on tumor lesions (26). In the third trial, patients with follicular lymphoma were vaccinated with PD-L1Long1 in combination with a PD-L2-derived peptide, and disease remission was detected in follow-up (27). Finally, PD-L1-vaccines were administered in combination with an IDO-derived peptide and a PD-1 blocking antibody to patients with metastatic malignant melanoma. The study showed a remarkable clinical effect, with a staggering 80% overall response rate (28). These four studies underlined the potential of a PD-L1-based peptide vaccine in solid as well as hematological cancers.

In the present study we report on a phase I first-in-man study in which ArgLong2 and PD-L1Long1 peptide vaccine combined with the adjuvant Montanide ISA-51 was performed in patients with MPN.

## Materials and methods

This phase I-II clinical vaccination trial was initiated at Herlev and Gentofte Hospital, Capital Region of Denmark. The trial aimed to determine the immunogenicity, clinical efficacy, and safety of a dual vaccination with ArgLong2 and PD-L1Long1 peptides, combined with Montanide ISA-51 as adjuvant, in patients with MPN. Patients were enrolled in the trial in the Departments of Hematology at Herlev and Gentofte Hospital, Copenhagen, and Zealand University Hospital, Roskilde, Denmark. The trial started on 7 October 2019 and the last patient received the last vaccine on the January 26^th^, 2021.

All participants provided written informed consent before trial enrollment. The protocol was approved by the Ethics Committee of the Capital Region of Denmark, the National Board of Health, and the Danish Data Protection Agency, and it was registered at https://www.clinicaltrials.gov (NCT04051307; date of registration: August 9, 2019).

We intended to include 24 patients in the trial and planned for the possibility of including an additional 24 patients if two or more patients from the first cohort showed a clinical response. However, due to the COVID-19 pandemic this could not be met. The main inclusion criteria were a diagnosis of ET or PV, according to WHO criteria (29). A full list of the inclusion and exclusion criteria is shown in **Supplementary Figure 1**. Patients could receive concurrent treatments with IFN-α, HU, or Anagrelide (ANA) in any combination, but no other anti-neoplastic or anti-MPN treatments were permitted.

To evaluate clinical responses, we applied the response criteria for PV and ET (30). A 10% reduction of the allele burden, based on a validated, in-house qPCR method, was defined as a response.

### Vaccine composition and treatment schedule

Patients were vaccinated with 200 µg of ArgLong2 (ARG1_169-206_), a 38-aa peptide (ISAKDIVYIGLRDVDPGEHYILKTLGIKYFSMTEVDRL), and 100 µg of PD-L1Long1 (PD-L1_19-27_), a 19-aa peptide (FMTYWHLLNAFTVTVPKDL). The peptides were provided by Polypeptide (Strasbourg, France).

The two vaccines were administered at the same time. Briefly, the peptides were individually dissolved in 500 µg sterile water/DMSO and emulsified with 500 µl Montanide ISA-51, just prior to administration. The vaccines were administered subcutaneously, one in each shoulder, every two weeks. Patients received six treatments (i.e., 12 vaccinations) over 12 weeks. After these first six treatments, treatment was paused for 1-3 months; then, six more treatments were given, again at two-week intervals. An additional six treatments could be scheduled for patients that showed a response. The entire treatment plan is shown in **Supplementary Figure 2**.

### Adverse events, safety, and toxicity evaluations

Adverse events were assessed according to the Common Terminology Criteria for Adverse Events, version 5.0. All patients were evaluated prior to inclusion with a medical examination by the treating physician. This included spleen palpation (no ultasonography nor computed tomography was performed), blood sample analyses (Hemoglobin, leukocyte differentiation count, platelets, IgG, IgA, IgM, Hematocrit, Bilirubin, Potassium, Sodium, Creatinine, albumin, uric acid, lactate dehydrogenase, Alkaline Phosphatase, Alanine transaminase, amylase, bilirubin, D-dimer, ionized calcium, C-Reactive Protein, Thyrotropin, thyroxin, Luteinizing Hormone, Adrenocorticotropic Hormone, Cortisol, Hepatitis B, hepatitis C (IgG), HIV, HTLV-1(IgG), IgG and IgM for Cytomegalovirus (CMV), Epstein-Barr Virus (EBV) and toxoplasmosis) and an electrocardiogram. According to the treatment plan, patients were evaluated at inclusion, during treatment pause, and at the end of the trial (EOT). At every vaccine treatment, an investigator recorded the patient’s symptoms, and when necessary, conducted a medical examination and evaluation. Bone marrow biopsies were acquired from patients before trial entry and at end of the trial. Histopathological evaluation of nonblinded biopsies was performed by a trained hematopathologist.

### DNA analyses with digital droplet polymerase chain reaction

We analyzed patient DNA samples for mutations with the droplet digital PCR (ddPCR) method, on a QX100 system (Bio-Rad, Hercules, California, USA), according to manufacturer instructions. Briefly, we mixed 10 µl of 2x digital PCR Supermix for probes (Bio-rad), 2 µl primer/probe mix, 3 µl nuclease-free water, and 5 µl DNA (20 ng/µl), for a total volume of 20 µl. Droplets were generated on a QX100 Droplet Generator System (Bio-rad). The polymerase chain reaction (PCR) was performed with an initial stage at 95°C for 10 min, followed by 43 cycles of 94°C for 30 sec and 57°C for 60 sec, then a final stage at 98°C for 10 min. PCR was carried out on an Applied Biosystems Veriti 96-Well Thermal Cycler (Thermo Fisher, Waltham, MA, USA). Droplets were subsequently quantified on a QX100 Droplet Reader (Bio-rad) and analyzed with Quantasoft™ Analysis Pro software.

The *JAK2V617F* primer/probe assay included a forward primer: GCTTTCTCACAAGCATTTG, a reverse primer: GCATTAGAAAGCCTGTAGTTTTA, and two probes: Fam-TCGTCTCCACAGAaACATACTCCATGAGACGA-BHQ1 (mutant c.1849G>T) and Hex-TCGTCTCCACAGACACATACTCCATGAGACGA-BHQ1 (wildtype).

### Next generation sequencing

Next generation sequencing was performed using the Illumina Ampliseq Myeloid panel including 40 genes (*ABL1, ASXL1, BCOR, BRAF, CALR, CBL, CEBPA, CSF3R, DNMT3A, ETV6, EZH2, FLT3, GATA2, HRAS, IDH1, IDH2, IKZF1, JAK2, KIT, KRAS, MPL, MYD88, NF1, NPM1, NRAS, PHF6, PRPF8, PTPN11, RB1, RUNX1, SETBP1, SF3B1, SH2B3, SRSF2, STAG2, TET2, TP53, U2AF1, ZRSR2, WT1*). Genomic DNA was purified from peripheral blood at baseline and 9 months after the first vaccination. Libraries were prepared using the Ampliseq for Illumina Myeloid Panel protocol, and 2 × 150 bp paired-end sequencing was done on the NextSeq 500 platform (Illumina^®^ Inc, San Diego, CA, USA). The Illumina Sequencing Analysis Viewer (SAV) software was used for quality control of the sequencing runs. Alignment of sequencing data to the human reference genome (GRCh37/hg19) and variant calling of mapped reads were performed in CLC Genomics Workbench software v.22. The VarSeq™ software v.2.2.4 (Golden Helix, Inc., Bozeman, MT, USA) was applied for annotation and filtering of variants. Variants with coverage <100x, a variant allele frequency (VAF) <1%, and germline, introns, and SNPs with minor allele frequency >1% (ExAC variant frequencies, Broad Institute, MA, USA) were excluded from further analysis.

### Isolation of bone marrow and peripheral blood

For peripheral blood mononuclear cell (PBMC) isolation, blood samples were obtained and cryopreserved as previously reported (31) at baseline, after three vaccinations, after six vaccinations (during the treatment pause), after seven vaccinations, after nine vaccinations, and at EOT. Heparinized bone marrow aspirations (10 mL in a heparinized tube) were obtained at baseline and at the end of the trial. Ortho-Lysing Buffer diluted 10× in H_2_0 was added to half of the sample, followed by centrifugation and incubation for 15 minutes in the dark. The other half of the sample was handled and cryopreserved following the same procedure as for PBMCs.

### Delayed-type hypersensitivity and skin-infiltrating lymphocytes

To assess the presence of PD-L1 and ARG1-specific lymphocytes, we performed a delayed-type hypersensitivity (DTH) test. DTH tests were assessed at baseline and at EOT. Briefly, we administered intradermal injections of the two peptides, without the adjuvant, at the lower back. The peptides were dissolved in sterile water and DMSO. We also administered a control injection, which included water and DMSO, but no peptide. At 48 h after the injection, skin reactions (induration) were measured; additionally, at the sites of ArgLong2- and PD-L1long1 injections, punch biopsies were acquired and cut into fragments. To identify fragments that contained skin-infiltrating lymphocytes (SKILs), fragments were cultured in 24-well plates in RPMI-1640 with 10% human serum and 100 U/mL interleukin-2 (IL-2), with penicillin, streptomycin, and fungizone, for 3–5 weeks to allow SKIL outgrowth. Every second or third day, half the medium was replaced with fresh medium containing IL-2, penicillin, streptomycin, and fungizone. After 3–5 weeks, SKILs were harvested, and a fraction was tested in ELISPOT assays. The remaining SKILs were cryopreserved.

### Interferon-γ ELISPOT assays *in vitro* and *ex vivo*

Immune responses were evaluated with *in vitro* and *ex vivo* IFN-γ ELISPOT assays. For *in vitro* ELISPOT assays, PBMCs were thawed and stimulated with the target epitope. The next day, PBMCs were stimulated with IL-2 (120 U/mL) and incubated for 12-14 days. The PBMCs were then counted and plated in ELISPOT wells. Cells were restimulated with or without the target peptide. All conditions were performed in triplicates. For *ex vivo* IFN-γ ELISPOT assays, cells were thawed, rested, then plated on ELISPOT plates. Cells were stimulated with or without the target epitope for 24-48 h to ensure antigen presentation.

*In vitro* ELISPOT assays were performed with a cell density of 2.5 ×10^5^ cells/well. *Ex vivo* ELISPOTs were performed with cell densities of 9 ×10^5^ cells/well, for PBMCs, and 6.8×10^5^ cells/well, for bone marrow mononuclear cells. Plates were analyzed with the ImmunoSpot Series 2.0 Analyzer (CTL, Shaker Heights, Ohio). Results were generated by subtracting the background obtained with negative controls. A detailed description of our setup was described previously (32). Statistical significance of ELISPOT responses was analyzed by the DFR method (33). For non-triplicate samples, responses were evaluated empirically and defined as true if the number of spots observed in the peptide stimulated wells were at least double of the spot counts in the control wells.

### Intracellular cytokine staining

To evaluate the phenotypes of T cells that responded to stimulation in IFN-γ ELISPOT assays, we conducted intracellular cytokine staining (ICS). The *in vitro* stimulation was similar to that described above in the IFN-γ ELLISPOT section. Briefly, after 12-14 days *in vitro culture*, PBMCs were restimulated with peptide. After 1 h, Brefeldin A was added to inhibit protein transport. After 4 additional hours of incubation, the cells were stained with T-cell surface markers CD4-PerCP (cat. 345770), CD8- FITC (cat. 345772), CD3-APC-H7 (cat. 560275) and a dead cell marker FVS510 (564406) (all from BD Biosciences). Samples were then fixed and permeabilized using eBioscience™ Fixation/Permeabilization buffers (eBioscience, cat. 00-5123-43, 00-5223-56) and stained with IFNg-APC (cat.341117, BD Biosciences), TNFa-BV421 (cat.562783, BD Biosciences) in eBioscience permeabilization buffer (eBioscience, cat. 00-8333-56) and analyzed on FACSCanto™ II (BD Biosciences) using BD FACSDiva software version 8.0.2 as described previously (32).

### RT-qPCR analysis of arginase-1 and PD-L1 expression

Analysis was performed on samples derived from patient PBMCs and bone marrow-derived MNCs isolated at baseline and end-of-trial. For the PBMCs, CD14 Microbeads (Miltenyi Biotec) were utilized to separate CD14+ and CD14- cells. Total RNA purification was performed using the RNEasy Plus Mini Kit (Qiagen) according to the manufacturer’s protocol. RNA concentration was measured on a NanoDrop2000 Spectrophotometer (Thermo Scientific). cDNA was synthesized using the iScript™ cDNA Synthesis Kit (Bio-Rad) based on 500 ng or 400 ng total RNA (PBMC-derived cells and bone marrow, respectively). RT-qPCR analysis was performed using the TaqMan Gene Expression Assay on a Roche LightCycler 480 instrument. The assay was performed in technical triplicates for all primers and the subsequent data analysis was performed as previously described using the ΔΔCt-method (34) with normalization of PD-L1 (Primer ID Hs01125296_m1) and ARG1 (Primer ID Hs00163660_m1) expression to the housekeeping gene POL2RA (Primed ID Hs00172187_m1) and to the baseline sample. Controls lacking reverse transcriptase during cDNA creation were included for primer validation.

### Phenotyping of PMBCs and bone marrow mononuclear cells

PBMCs collected at three time-points and bone marrow collected at 2 time-points were thawed and washed in preheated phosphate-buffered saline. Fc-receptors were blocked by incubating with human IgG (50 μg/ml), and dead cells were stained with the Fixable Near-IR Dead cell stain kit (Thermo-Fisher). After mixing, the cells were stained in the dark at 4°C for 20 min with fluorochrome-labeled antibodies (**Supplementary Figure 3**). Next, the cells were washed and analyzed with a NovoCyte Quanteon Flow Cytometer (Agilent, Santa Clara, CA). The gating strategy is described in **Supplementary Figure 4**. Data were analyzed with NovoExpress 1.5.1 software. All gates at baseline were applied to all timepoints. Illustrations were created with Graphpad Prism v 8.0 (GraphPad Software. Inc.).

### Statistical analysis

ELISPOT responses were analyzed with the distribution free resampling (DFR) method (33). DFR analyses were performed with the R statistical analysis program. Immune subsets at different time points were compared with the Wilcoxon matched-pairs signed-rank test. *P* values ≤0.05 were considered significant. Immune subset analyses were performed in Graphpad Prism v 8.0 (GraphPad Software. Inc.).

## Results

### Patient characteristics and clinical response evaluation

We evaluated bone marrow biopsies and blood samples from 12 patients before inclusion in the vaccination trial. Three patients did not meet the inclusion criteria and were excluded: One patient had patient had unmeasurable JAK2V617F and normal bone marrow and thus did not meet the diagnostic criteria, one patient had progressive disease and proceeded to alloHSC and one patient had additional findings in the bone marrow suggesting a more MDS-like disease and thereby did not meet the diagnostic criteria Thus, nine patients (5 male, 4 female, median age: 57 years, range: 46 to 72) with a median disease duration (time from diagnosis) of 2 years (range: 4 months to 10 years) were enrolled in the clinical study. The vaccines were administered over a period of six to nine months. Among the nine patients, eight received a minimum of 12 vaccines; one patient did not receive one ArgLong2 vaccine. One patient (#3) proceeded with an extra round of vaccinations, due to an apparent drop in the *JAK2*V617F allele burden, measured with qPCR. This drop could however not be confirmed by ddPCR. The primary end of trial (EOT) was defined as 12 treatments.

**Figure 1:**
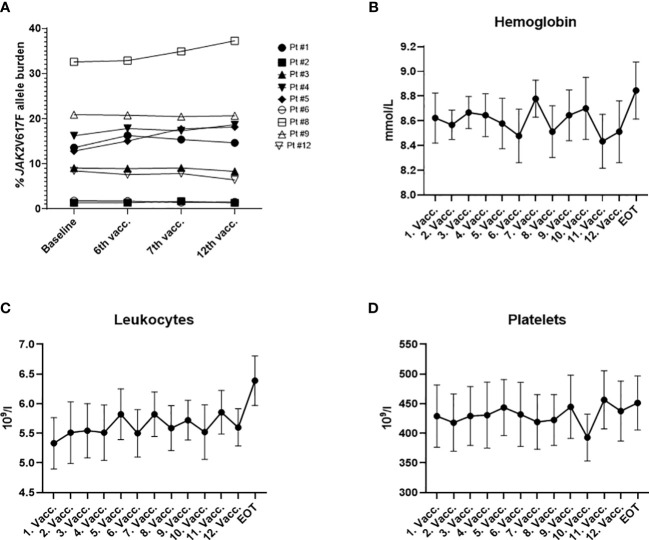
**(A)** *JAK2*V617F allele burden measured in the peripheral blood by digital droplet PCR during the study. **(B)** Cumulative change in hemoglobin (mmol/l) during the trial. **(C)** Cumulative change in leucocytes (×10^9^/l) during the trial. **(D)** Cumulative change in platelets (×10^9^/l) during the trial. **(B, C)** graphs depict the mean ± SEM.

At inclusion, all nine patients had a measurable *JAK2*V617F mutation burden, ranging from 1.36% to 32.58% (median 12.81%). Additionally, two patients harbored bystander mutations in the *DNMT3A* gene as determined by next generation sequencing. Seven patients were diagnosed with ET, and two patients were diagnosed with PV (29). At the time of inclusion seven out of nine patients received cytoreductive treatments for MPN: these treatments included IFN-α (N=5, Pegasys, 4 patients received 45 ugx1sc/week, 1 patient received 135 ug x 1 sc/every fourth week), ANA (N=1), and phlebotomy (N=1). The median platelet count for the patient cohort was 489 × 10^9^/L (range: 219 – 630 × 10^9^/L). The median hemoglobin count was 8.6 mmol/L (range: 7.8 – 9.7 mmol/L); the median hematocrit was 0.41 (range: 0.39 – 0.46) and the median leucocyte count was 5.2 × 10^9^/L (range: 3.5 – 8.2 × 10^9^/L). Patient baseline characteristics are summarized in **Table 1**. At the primary EOT, the *JAK2*V617F mutation burden ranged from 1.33% to 37.25% (median 14.64%; **Figure 1A**). Bone marrow samples and blood samples were evaluated to investigate any potential impact of the vaccines on hematological response and molecular response in addition to changes in disease phenotype. However, we did not identify any signs of neither a molecular (**Figure 1A**) nor a hematological response on average (**Figures 1 B–D**, **Supplementary Figure 5**). No alterations in bone marrow architecture and cellular composition were identified in any of the patients (data not shown).

**Table 1:** Patient baseline characteristics.

Characteristic	Patients
Sex	Female n= 4, Male n=5
Age at inclusion in years, median (min-max)	57 (46-72)
Duration of disease in years, median (min-max)	6.5 (2-26)
Diagnosis	ET n=7; PV n=2
Treatment	Pegylated Interferon-alpha n=5 No Treatment n=2 Anagrelide n=1 Phlebotomy n=1
Platelet count at inclusion, median (min-max)	489x10^9^/l (219x10^9^/l – 630x10^9^/l )
Hemoglobin at inclusion, median (min-max)	8,6 mmol/l (7,8mmol/l – 9,7mmol/l)
Leukocyte count at inclusion, median (min-max)	5,2x10^9^/l (3,5x10^9^/l – 8,2x10^9^/l)
Lactate dehydrogenase, median (min-max)	199 U/mL (172 U/mL - 224 U/mL)
Erythrocyte volume fraction, median (min-max)	41 % (39 % - 46 %)
MPN driver mutation	*JAK2*V617F n=9
% *JAK2*V617F VAF at inclusion, median (min-max)	12,81% (1,36% - 32,58%)

### Vaccine tolerability and safety

Vaccines were generally well tolerated: the majority of reported adverse events were grades 1 and 2 (**Table 2**). Injection site reaction (grade 2) was the most commonly observed adverse event and was reported by all the patients at least once during the study period. In two patients, injection site reactions remained visible for one year after the last vaccine (patients #4 and #5). Only one grade 3 adverse event was registered: one patient (# 9) had a vasovagal reaction immediately after the first PD-L1Long1 vaccination and did not receive the ArgLong2 peptide vaccine during the same hospital visit. However, the patient continued the treatments afterwards and received both vaccines, as scheduled, without similar reactions. There were two peculiar adverse events reported by the same patient during the trial: a change in taste (grade 1 dysgeusia) and a change of body odor (grade 1). The patient reported on this at the time of the last vaccine, but claimed that the changes started already after the first vaccine. The reactions were deemed to be related to the DMSO contained in the vaccine solution. Patient # 1 reported several reactivations of Herpes Simplex virus during the trial (at treatments 3, 5, 7, and 11). The patient had a history of Herpes Simplex infections, but the last reactivation had occurred several years before study inclusion.

**Table 2:** Reported adverse events.

Type	Number of patients	Grade 1	Grade 2	Grade 3
Change in body odor	1	1		
Diarrhea	1	1		
Dry skin	1	1		
Dysgeusia	1	1		
Edema limbs	1	1		
Eczema	1		1	
Fatigue	3	2	1	
Flu like symptoms	3	3		
Herpes simplex reactivation	1		1	
Headache	1	1		
Infection	3	1	2	
Injection site reaction	9		9	
Pain	2	2		
Palpitations	1	1		
Pruritus	2	2		
Rotator cuff injury	1	1		
Vasovagal reaction	1			1

### Immune responses

**Figure 2:**
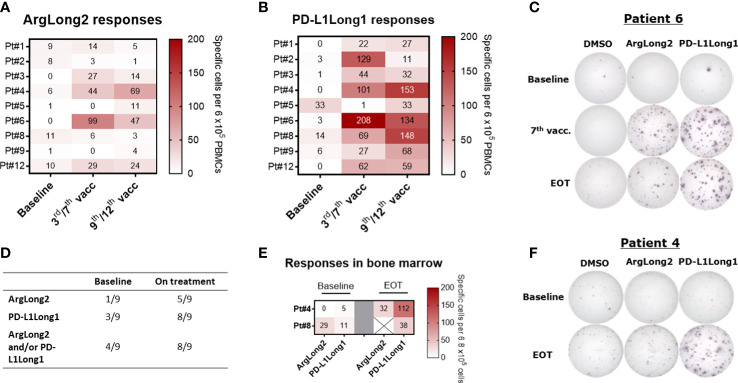
*Ex vivo* IFNγ ELISPOT responses in patient PBMCs and BMNC during treatment. **(A, B)** Heat map of *ex vivo* PBMC responses against the ARG1 **(A)**- and PD-L1**(B)**-derived peptide epitopes at baseline and during treatment. **(C)**: Representative well images of *ex vivo* IFNγ ELISPOT response against ArgLong2 and PD-L1Long1 in PBMCs from patient 6. **(D)** Summary of statistically significant PBMC responses from *ex vivo* IFNγ ELISPOT assay. **(E)** Heat map of *ex vivo* responses against the ARG1- and PD-L1-derived peptide epitopes in BMNC of patient 4 and 8 as determined by IFNγ ELISPOT. **(F)** Representative well images of *ex vivo* ELISPOT response in BMNC from patient 4. The number of peptide-specific cells in ELISPOT assay was calculated by subtracting the mean number of spots in the control wells from the mean number of spots in the peptide-stimulated wells. PBMCs were plated at a density of 9×10^5^ cells/well and BMNCs at 6.8×10^5^ cells/well.

To assess the vaccine induced immune responses, we analyzed PBMCs and BMNCs for T-cell responses against the two peptides used in the trial by *ex vivo* IFN-γ ELISPOT assay (**Figure 2**). We detected a spontaneous response against the ARG1- and the PD-L1-derived peptides in PBMCs from one out of nine and from three out of nine patients at baseline respectively. During treatment, the responses against the ARG1-derived and PD-L1-derived peptides were detected *ex vivo* in PBMCs from five and eight patients respectively (**Figures 2A–D**). In patient 5, we did not observe a significant *ex vivo* PBMC response during treatment, even though the patient’s PBMCs displayed a spontaneous T-cell response at baseline. *Ex vivo* IFN-γ ELISPOT was performed using bone marrow mononuclear cells (BMNC) from patients 4 and 6, and cells from both patients showed vaccine-specific T cells after treatment with an apparent increase in responses in EOT samples compared to baseline (**Figures 2E, F**). Due to low viability of the isolated BMNC, we were not able to perform a similar assay on BMNCs from the remaining seven patients.

**Figure 3:**
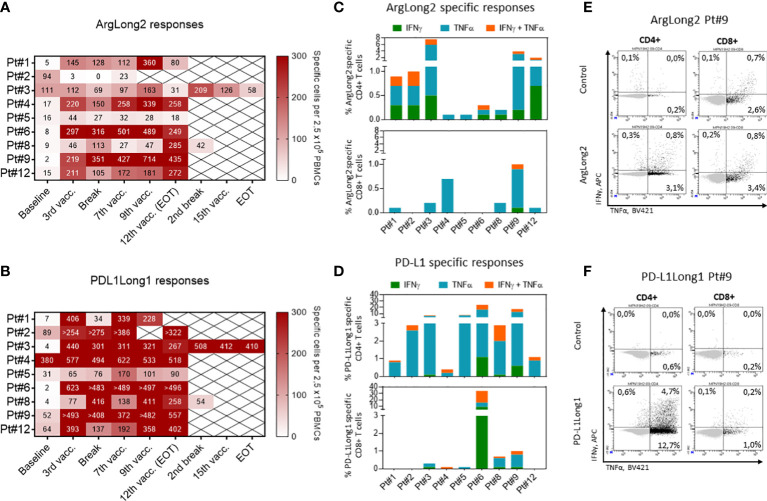
Responses against ARG1- and PD-L1-derived peptides in PBMCs. **(A, B)**: Heat maps depicting PBMC responses against the ARG1-**(A)** and PD-L1-**(B)** derived peptide epitopes as measured by *in vitro* IFNγ ELISPOT. The number of peptide-specific cells was calculated by subtracting the mean number of spots in the control wells from the mean number of spots in the peptide-stimulated wells. PBMCs were plated at a density of 2.5×10^5^ cells/well. **(C, D)** The phenotype of vaccine specific T cells in *in vitro* cultured PBMCs of treated patients as determined by intracellular staining for IFNγ and TNFα production in CD4+ (top) and CD8+ (bottom) T cells in response to ARG1-**(C)** and PD-L1-**(D)** derived peptide stimulation. Bars represent peptide specific response after background subtraction. **(E, F)** Representative dot plots of IFNγ and TNFα cytokine production by CD4+ (left) and CD8+ (right) T cells in response to ArgLong2 **(E)** and PD-L1Long1 **(F)** peptide restimulation as compared to unstimulated control in *in vitro* cultured PBMCs from patient 9.

In an addition to *ex vivo* assays, we performed an *in vitro* IFN-γ ELISPOT and intracellular cytokine staining (ICS) on *in vitro* pre-stimulated PBMCs to improve the detection of the treatment induced response and phenotypically characterize the vaccine reactive T cell populations (**Figure 3**; **Supplementary Figures 6 D–F**). At baseline, we detected spontaneous responses against the ARG1-derived peptide in the PBMCs of six of nine patients, albeit most of the responses were low in magnitude similarly with the *ex vivo* ELISPOT results. During the vaccine trial, all patients but patient 2, demonstrated an enhanced ARG1-specific T cell response with a clear increase in ArgLong2 response magnitude already after 3^rd^ vaccination (**Figure 3A**; **Supplementary Figures 6A, C**). PBMCs from six out of nine patients displayed a spontaneous response against the PD-L1-derived peptide at baseline, with a majority of the baseline responses being low in magnitude (**Figure 3B** and **Supplementary Figure 6B**). Only PBMCs from patient 8 did not display a spontaneous response against neither the PD-L1 nor ARG1 epitopes at baseline. PBMCs from all patients displayed greatly enhanced T-cell responses against the PD-L1-derived epitope after 3 vaccinations (**Figure 3B**; **Supplementary Figures 6B, C**).

In ICS we chose to analyze PBMCs collected at time points in which the individual patients showed the strongest immune response as detected by *in vitro* ELISPOT (**Figures 3A, B**). We detected that the majority of the vaccine specific T cells were producing TNF-α and/or IFN-γ in response to peptides used in the vaccination. Responses against ArgLong2 were identified in all nine patients, though primarily in the CD4^+^ T-cell compartment with lower incidence of CD8^+^ T-cell responses detected in 6 out of 9 patients (**Figures 3C, E**). Similarly, the analyses confirmed that all 9 patients generated CD4^+^ T-cell response against PD-L1-derived peptide and 6 out of 9 patients also displayed a lower magnitude of CD8^+^ T cell response against the same peptide (**Figures 3D, F**).

Induction and expansion of vaccine specific T cell responses was also evaluated by delayed type hypersensitivity (DTH) testing. Six out of nine patients provided informed consent for this analysis. Baseline DTH biopsies collected from four out of six patients could not be expanded to obtain skin infiltrating lymphocytes (SKILs) cultures. In baseline SKILs samples obtained from patients 2 and 9 we could only detect a significant response against the ARG1-derived peptide in IFNγ ELISPOT in patient 9 (**Supplementary Table 1**). At EOT we expanded SKILs from biopsies collected from all six patients and in 5 out 6 analyzed patients, the SKILS exhibited vaccine-specific responses against one or both peptides as determined by IFNγ ELISPOT signifying expansion and target epitope dependent homing of the vaccine induced T cells (**Supplementary Table 1**). None of the patients displayed a visible skin reaction at baseline, however at EOT (corresponding to the expanded SKILs samples), all patients except patient 1 showed skin reactions with redness and swelling to various degrees at all injected DTH sites.

### Phenotypic characterization of PBMCs during treatment

We investigated the peripheral blood of the patients for changes in composition of immune cell subsets during the vaccination trial. We performed fluorescence-activated cell sorting (FACS) on isolated PBMC at three time-points: baseline, treatment pause (after 6 treatments), and EOT. Bone marrow aspirations acquired at baseline and at EOT were also analyzed.

**Figure 4:**
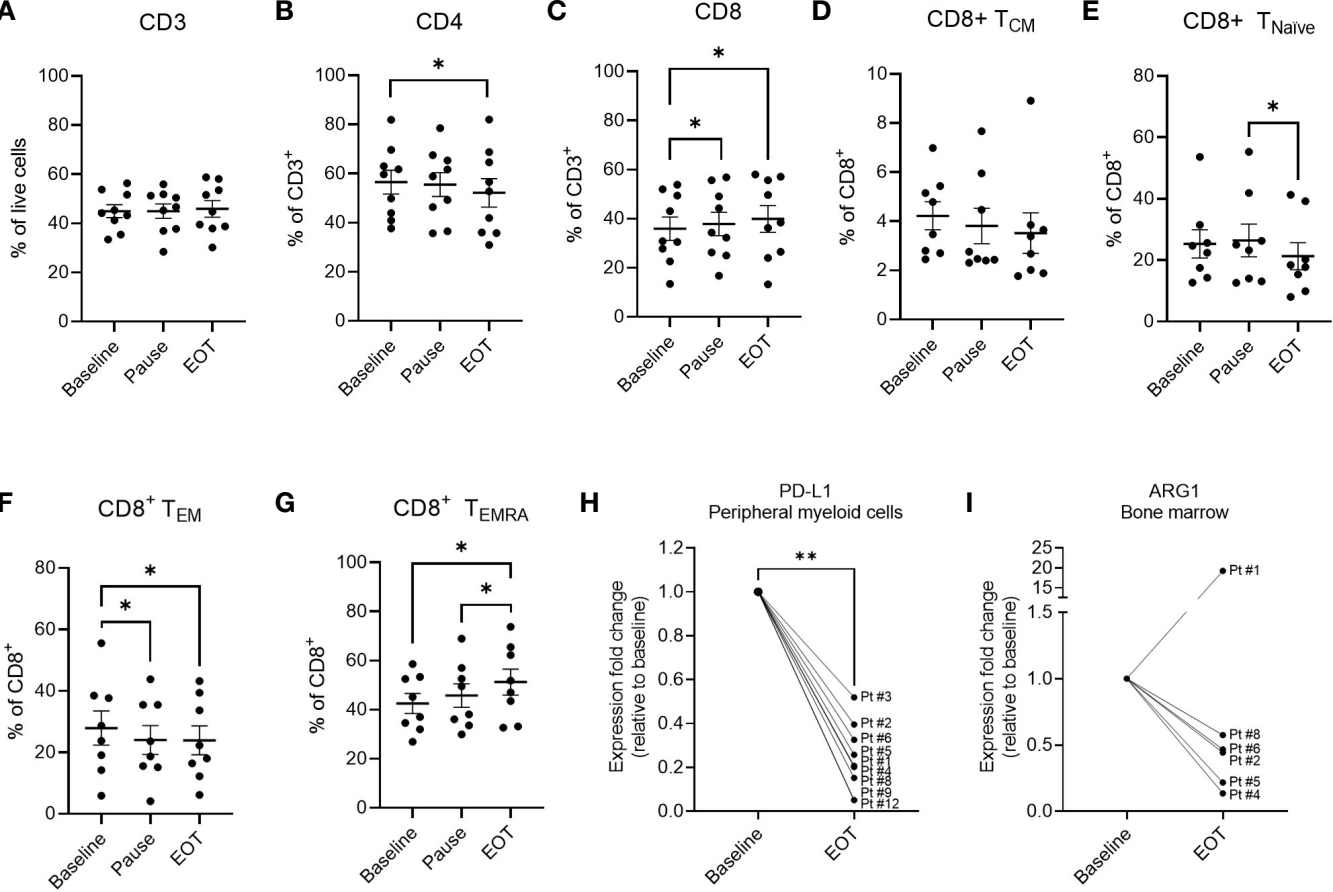
Phenotypic marker expression in PBMCs and BMNCs at baseline and during vaccination trial. Percentage of CD3+ **(A)**, CD4+ **(B)** and CD8+ **(C)** cells out of live cells in PBMCs of treated patients analyzed by flow cytometry. **(D–G)** changes in the subpopulations of CD8+ T cells in the PBMCs of treated patients. **(D)** Fraction of CD8+ central memory (CM) T cells defined as CD3^+^CD8^+^CD45RA^-^CCR7^+^represented as percentage out of CD8+ population. **(E)** The fraction of CD8+ naïve T cells defined as CD3^+^CD8^+^CD45RA^+^CCR7^+^ out of CD8+ cells. **(F)** The fraction of CD8+ effector memory (EM) cells defined as CD3^+^CD8^+^CD45RA^-^CCR7^-^ out of total CD8+ cells **(G)** The fraction of CD8+ T_EMRA_ cells defined as CD3^+^CD8^+^CD45RA^+^CCR7^-^ represented as percentage out of CD8+ population. **(H)** Changes in the expression of PD-L1 as determined by RT-qPCR in the CD14+ myeloid cells in the peripheral blood of patients at baseline and end of treatment. **(I)** Changes in the expression of ARG1 as determined by RT-qPCR in the total BMNCs of patients at baseline and end of treatment. Statistical analysis was performed using the Wilcoxon signed-rank test, *- p ≤0.05, **- p ≤0.01. A-G graphs represent mean ± SEM.

The fraction of CD3^+^ T cells in PBMCs remained unchanged throughout the study period (**Figure 4A**). However, the percentage of CD4^+^ T cells decreased significantly from baseline to EOT, and accordingly the fraction of CD8^+^ T cells increased significantly during treatment (**Figures 4B, C**). The significant increase in the percentage of CD8^+^ T cells was detectable already after six treatments. We further analyzed the changes in the CD8^+^ T cell subsets and observed a significant decrease and increase in the CD8^+^ T cell T_EM_ and T_EMRA_ subpopulations respectively by the end of the treatment (**Figures 4F, G**). A significant decrease in T_EM_ cells was already detectable at the treatment pause. T_EMRA_ cells increased from baseline to EOT. Additionally, while no changes were seen in the T_CM_ population, a significant decrease in CD8^+^ T_Naïve_ cells was seen (**Figures 4D, E**). The CD4^+^ T-cell subsets remained unchanged, including central memory (T_CM_), effector memory (T_EM_), T_Naive_, and T_EMRA_ cells (**Supplementary Figure 7**). PD-1 expression on CD3^+^ T cells as well as CD4^+^ and CD8^+^ T cell subsets remained unchanged (**Supplementary Figures 8A–C**). No changes in levels of regulatory T cells, natural killer (NK) cells, or B cells were observed during treatment (data not shown). CD16^dim^CD56^hi^ NK cells decreased significantly during the trial. MDSCs, defined as HLA^-^DR^-^CD33^+^CD14^+^ cells, monocytes, and dendritic cells remained stable during treatment. CD3^+^-, CD4^+^-, CD8^+^- and NK-cells in the bone marrow remained stable during treatment (data not shown).

### Changes in ARG1 and PD-L1 expression in treated patients

Changes in the expression of PD-L1 were assessed by flow cytometry and no alterations of PD-L1 expression on the myeloid cells (CD3^-^CD19^-^CD56^-^ cells) were observed during the trial (**Supplementary Figure 8D**). Changes in PD-L1 and ARG1 expression were also evaluated in PBMCs (n=9) and BMNCs (n=6) of the treated patients using RT-qPCR. Interestingly, a significant decrease in the PD-L1 mRNA expression was seen in CD14^+^ myeloid cells in the peripheral blood from baseline to end-of-treatment (**Figure 4H**). Decrease in PD-L1 expression was also detected in CD14^Neg^ cell fraction in 6 out of 7 evaluated patient samples (**Supplementary Figure 8E**). Interestingly, while no ARG1 expression was detected in the peripheral blood in neither CD14^+^ nor CD14^Neg^ cell fractions, a clear decrease in the ARG1 expression was detected in BMNCs of 5 out of 6 evaluated patients (**Figure 4I**). Contrary to ARG1, expression of PD-L1 was only detected in BMNCs of one patient (#5) and an increase in PD-L1 expression was seen in this sample (**Supplementary Figure 8F**). These results suggest a potential differential expression of ARG1 and PD-L1 between the peripheral blood and the bone marrow compartments in patients with MPN.

## Discussion

We successfully conducted a First-in-Man trial with an ARG1-derived peptide vaccine combined with a PD-L1-derived peptide vaccine in patients with MPN. We found that the vaccines were safe as vaccine related adverse events of only grade 1 and 2 were observed. The most common adverse event was development of granulomas at the injection site during treatment, which was observed in all patients and was related to the adjuvant (35). A single grade 3 vasovagal adverse event was reported, but was concluded to be unrelated to the vaccines.

We have previously demonstrated that ARG1- and PD-L1-specific T cells can directly target immunosuppressive cells (18,) (20,) (23), and we therefore hypothesized that a boost in the ARG1- and PD-L1-specific T-cell responses should lead to a reduction in the number of immunosuppressive cells in MPN which in turn could increase the tumor specific T-cell responses in treated patients. Our previous studies (12, 24) have shown that among patients with MPN patients with myelofibrosis display a significantly reduced T-cell response to ARG1- and PD-L1-derived peptide epitopes as compared to patients with ET (12, 36). This could reflect a dysregulation in the immune system in patients with advanced disease. In the current study, in line with our hypothesis, we observed an increase in specific T-cell responses against both the ARG1- and the PD-L1-derived epitopes in vaccinated patients as detected by *ex vivo* and *in vitro* IFN-γ ELISPOT assays (**Figures 2**, **3**). Both vaccines generated clear specific immune responses already after 3 vaccinations which appeared to be maintained until the end of the study. Interestingly, the majority of the vaccine specific T-cell responses were detected among the CD4^+^ T cells for both vaccine epitopes (**Figures 3C, D** This is in part due to the use of long peptide epitopes (38aa and 19aa) for the T cell stimulation., The processing of long peptides into HLA_I restricted epitopes requires uptake and intracellular processing of long peptides which may delay the presentation of class I epitopes in comparison to class II. As the standard duration of the ICS assay is only five hours, this might not be enough time for optimal processing and presentation of the HLA-I epitopes. A significant overall increase in the number of CD8^+^ T cells was seen in the peripheral blood during treatment. Among the CD8^+^ T cell subsets, the terminally differentiated T_EMRA_-cell population increased, and the CD8^+^ T_EM_-cell population significantly decreased suggesting that the vaccines boosted the CD8-mediated effector arm of the immune system.

During the treatment, T cell responses against both vaccination peptides were also observed among the infiltrating T cells in the DTH skin biopsies and bone marrow as detected by *ex vivo* and *in vitro* IFN-γ ELISPOT assays. This shows that T cells reactive against ARG1- and PD-L1-derived epitopes were able to home to tissues with increased presentation of these epitopes. Interestingly, in addition to induction of vaccine specific T cell responses, we were also able to show a decrease in ARG1 and PD-L1 expression as detected by RT-qPCR in bone marrow and peripheral blood respectively. These findings indicate the immunomodulatory capacity of the ARG1 and PD-L1 specific T cells *in vivo*. In the present study the data could suggest, that ARG1 specific T cells home to the bone marrow and kill ARG1-expressing target cells. However, it is still unknown if transformed myeloid cells in the bone marrow produce more ARG1 than non-transformed myeloid cells. PD-L1-specific CD8^+^ T cells can directly kill PD-L1-expressing cells (21). However, it should be taken into account that PD-L1-specific CD4^+^ T cells may increase the fraction of PD-L1^+^cells, due to the local production of pro-inflammatory cytokines (37, 38).

We were unable to observe a clinical effect in the treated patients within the trial period. All patients except patient 5 had peripheral blood values that remained stable during treatment. Likewise, bone marrow histology and the *JAK2*V617F allele burden remained generally unchanged for seven out of nine patients. Two patients (#5 and #8) showed an increasing allele burden over the course of the treatment (**Figure 1A**). The lack of clinical response could be explained by the low number of recruited patients for the trial, as we only included 9 of the intended 24 patients. In MPN the measured JAK2V617F mutational burden is very high which translates into a high tumor burden that might be impossible for the cellular immune system to control. It is noteworthy that patient 5 was identified as having MPN through participation in the population study termed the Danish General Suburban Study (GESUS) (39), In January 2018 the patient had a *JAK2*V617F allele burden of 4%, normal blood cells counts, as well as a normal bone marrow. Accordingly, the patient had *JAK2*V617F clonal hematopoiesis of indeterminate potential (CHIP). Nineteen months later, the patient was diagnosed with ET, and was in an early MPN-disease stage at the time of inclusion in the present study (after four additional months). Despite the early disease stage, the patient did not exhibit an *ex vivo* and only a limited *in vitro* ELISPOT response against either of the vaccine-derived epitopes during the treatment. The *JAK2*V617F allele burden in this patient increased from 12% to 17% during the trial, and platelet counts increased from 523 to 706 × 10^9^/l. These findings, including PD-L1 expression in the bone marrow (**Supplementary Figure 8F**), point towards a severe dysregulation of the immune system in this patient. We have earlier shown in another trial of therapeutic cancer vaccines in MPN, that patients with PMF show weaker immune responses to the vaccine compared to patients with ET (31). However, the patient mentioned above demonstrates that an exhausted immune response is not only restricted to patients with advanced MPN disease.

In future studies, it would be intriguing to vaccinate individuals with CHIP, as these patients have a very low tumor burden compared to patients with overt MPN. Otherwise, it will be necessary to combine the vaccines used in the present trial with other agents like an immune-checkpoint inhibitor. To date, treatments with the PD-1 checkpoint inhibitor, Pembrolizumab, have been unsuccessful in patients with MPN (40). However, a case study of a 71-year-old man with ET treated with the PD-1 checkpoint inhibitor, pembrolizumab, for a PD-L1-positive lung adenocarcinoma reported a decrease in elevated platelet counts and a dramatic decrease in the *JAK2*V617F allele burden after 17 months of pembrolizumab treatment (41). The immune-induced killing of adenocarcinoma cells in the patient may have re-activated the MPN-specific T-cell activity in the patient as the *JAK2*V617F mutation is also found in some carcinoma cells. Overall, in late-stage MPN, a PD-1:PD-L1 blockade may be hampered by the general deregulation and exhaustion of the immune system (4), which is characterized by a down-regulation of HLA molecules and severe gene dysregulation of proteins involved in inflammation (42, 43). Thus, the increased frequency of vaccine specific T cells combined with the overall increase in CD8^+^ cells observed in this study might be enhanced by checkpoint blockade. Thus, this combination might evoke a stronger immune response against both tumor cells and regulatory cells. Our group has previously shown that both the *JAK2*V617F mutation and the *CALR* frameshift mutation lead to generation of immunogenic peptides, and patients with these mutations spontaneously harbored specific T-cell responses against these epitopes (44–47). Combination therapy with IFN-α/α2 might also be an appealing option to further boost the immune activation. It should be noted that in our study five patients received IFN-α/α2 and no additional side effects were seen in this group, compared to patients not receiving IFN-α/α2.

In conclusion, we have tested a dual ARG1/PD-L1 peptide vaccination for the first time in humans. We have demonstrated that the combination of the two vaccines was safe and tolerable. We observed an induction or enhancement of vaccine-specific T-cell responses in all nine patients. Vaccine-specific T cells were also detected in the bone marrow after treatment and a decrease in ARG1 and PD-L1 expression was seen in bone marrow and peripheral blood respectively. Immune phenotyping of PBMCs showed a significant increase in the proportion of CD8^+^ T cells after the vaccination and an indication of activated CD8^+^ memory T cell subtypes. We did not observe an immediate effect on the *JAK2*V617F allele burden within the trial period. However, late onset disease regression as evidenced by complete remission after the finalization of the vaccination treatment was recently observed in two patients with follicular lymphoma treated in a similar trial with a dual PD-L1/PD-L2-derived peptide vaccine (27). Hence follow up of patients in the trial will be performed by annual measurements of peripheral blood counts and the *JAK2*V617F allelic burden, which will allow us to identify any late-onset responders among the vaccinated patients.

## Data availability statement

The datasets presented in this article are not readily available because of the Danish Law on data protection and the GDPR rules. Requests to access the datasets should be directed to the CCIT-DK office.

## Ethics statement

The protocol was approved by the Ethics Committee of the Capital Region of Denmark, the National Board of Health, and the Danish Data Protection Agency, and it was registered at www.clinicaltrials.gov (NCT04051307). The patients/participants provided their written informed consent to participate in this study.

## Author contributions

All authors contributed to the manuscript in regards to either conceptualizing and designing the trial or performing research or collecting, analyzing and interpretating data. All authors took part in writing or revision of the manuscript.

## Supplementary material

Supplementary Figure 1 Patient inclusion and exclusion criteria.

Supplementary Figure 2 Gant chart of the treatment and sample collection schedule.

Supplementary Figure 3 Antibodies used in phenotypic PBMC characterization using fluorescence-activated cell sorting.

Supplementary Figure 4 Fluorescence-activated cell sorting gating strategies, applied with NovoExpress 1.5.1 software.

Supplementary Figure 5 Analysis of mean lactate hydrogenase (left) and erythrocyte volume fraction (right) in the treated patients (n=9) during the study. Error bars depict the standard error of the mean.

Supplementary Figure 6 Summary of statistical significance analysis of *in vitro* and *ex vivo* IFNγ ELISPOT responses to ArgLong2 and PD-L1Long1 during the study shown in and 3. **A**, **B:** Statistical analysis summary of *in vitro* IFNγ ELISPOT responses to ArgLong2 **(A)** and PD-L1Long1 **(B)**. **C**. Summary of statistically significant results from ELISPOT assays showing the number of patients with responses to either one epitope or both. **D**, **E:** Statistical analysis of *ex vivo* ELISPOT responses to ArgLong2 **(D)** and PD-L1Long1 **(E)**. **F**: Overview of statistically significant results from ELISPOT assays showing the number of patients with responses to one or both vaccine peptides. * indicates statistical significance, based on the DFR method (33); ns-nonsignificant response; DR (for non-triplicate samples only): empirical response defined as true, when at least twice the number of spots were observed in the peptide wells, compared to the number in control wells.

Supplementary Figure 7 Characterization of CD4^+^ T-cell subsets in PBMCs of treated patients. **A.** Fraction of CD4+ central memory (CM) T cells defined as CD3^+^CD4^+^CD45RA^-^CCR7^+^. **B.** The fraction of CD4+ naïve T cells defined as CD3^+^CD4^+^CD45RA^+^CCR7^+^. **C.** The fraction of CD4+ effector memory (EM) cells defined as CD3^+^CD4^+^CD45RA^-^CCR7^-^. **D.** The fraction of CD4+ T_EMRA_ cells defined as CD3^+^CD4^+^CD45RA^+^CCR7^-^. Statistical analysis was performed using the Wilcoxon signed-rank test. Graphs represent mean values ± standard error of the mean.

Supplementary Figure 8 Expression of PD-1 on CD3+ **(A)**, CD4+ **(B)**, CD8+ **(C)** cells in the peripheral blood as detected by flow cytometric analysis at baseline and during the trial. Graphs represent mean values ± the standard error of the mean. **D:** FACS analysis of PD-L1 expression on CD19^−^CD3^−^CD56^−^ myeloid cells at baseline and during the vaccination trial. **E:** PD-L1 expression by CD14 negative cells in PBMCs of treated patients before and after treatment as measured by RT-qPCR. **F:** PD-L1 expression in BMNCs of patient 5 before and after treatment as measured by RT-qPCR.

Supplementary Table 1 Summary of IFNγ ELISPOT assay results for peptide specific responses in skin infiltrating lymphocytes (SKILS) expanded from delayed type hypersensitivity test in patients with MPN, before and after the trial (EOT). Intradermal injections of the two peptides, dissolved in sterile water and DMSO were biopsied 48 hours after administration. SKILS were expanded in media containing IL2 and harvested. ***** = significant (DFR) immune response detected; **ns** = No significant (DFR) immune response detected; **N/A** – not available due to lack of outgrowth of SKILS.
