# Supplementary material for: An arginase1- and PD-L1-derived peptide-based vaccine for myeloproliferative neoplasms: A first-in-man clinical trial
Source: Front Immunol. 2023 Feb 23;14:1117466. doi: 10.3389/fimmu.2023.1117466 (PMC9996128; doi:10.3389/fimmu.2023.1117466)
Supplement: Supplementary Figure 1 — Patient inclusion and exclusion criteria. [file Presentation_1.pptx]

## Slide 1
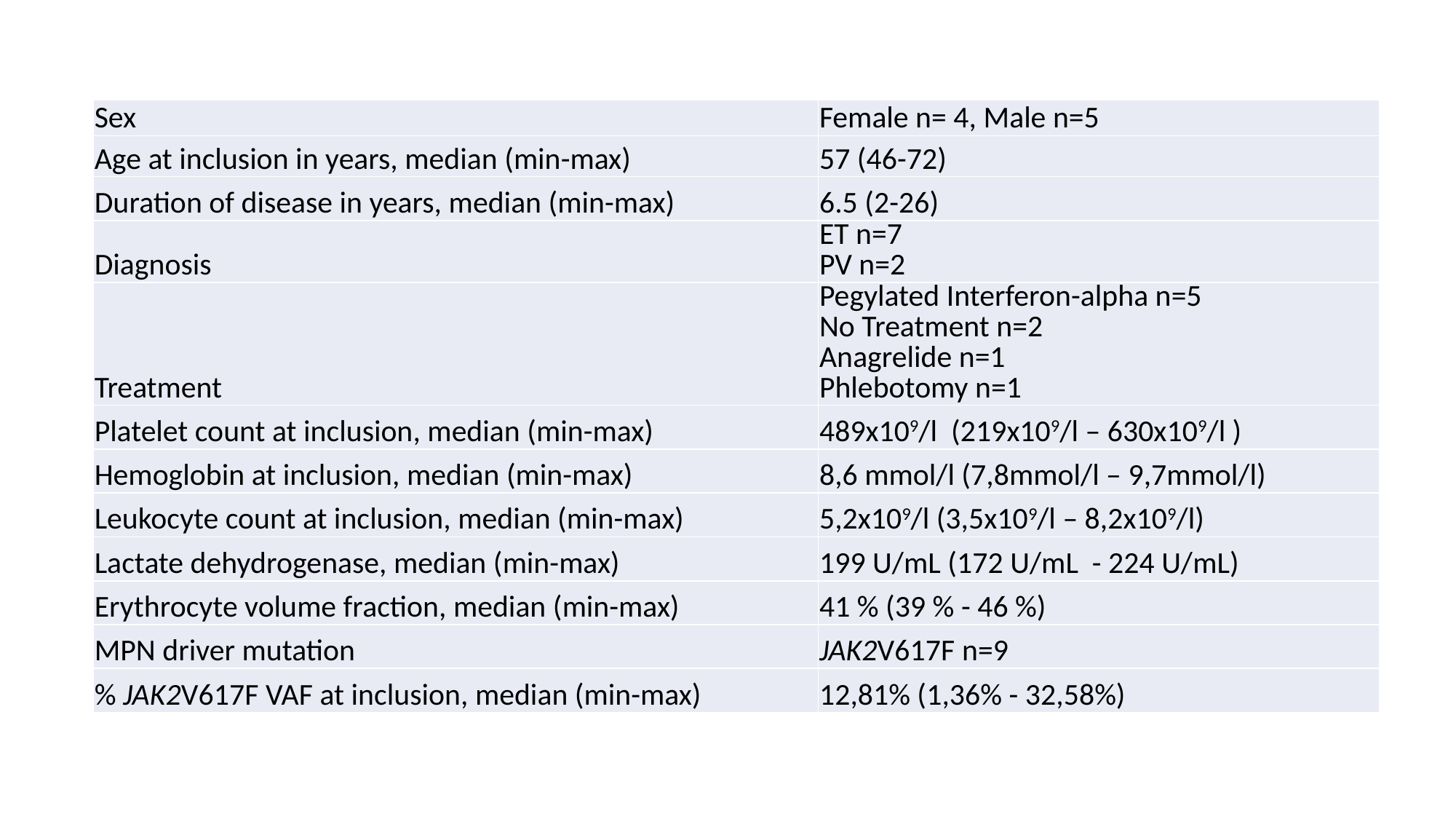

| Sex | Female n= 4, Male n=5 |
| --- | --- |
| Age at inclusion in years, median (min-max) | 57 (46-72) |
| Duration of disease in years, median (min-max) | 6.5 (2-26) |
| Diagnosis | ET n=7 PV n=2 |
| Treatment | Pegylated Interferon-alpha n=5 No Treatment n=2 Anagrelide n=1 Phlebotomy n=1 |
| Platelet count at inclusion, median (min-max) | 489x109/l (219x109/l – 630x109/l ) |
| Hemoglobin at inclusion, median (min-max) | 8,6 mmol/l (7,8mmol/l – 9,7mmol/l) |
| Leukocyte count at inclusion, median (min-max) | 5,2x10^9^/l (3,5x10^9^/l – 8,2x10^9^/l) |
| Lactate dehydrogenase, median (min-max) | 199 U/mL (172 U/mL - 224 U/mL) |
| Erythrocyte volume fraction, median (min-max) | 41 % (39 % - 46 %) |
| MPN driver mutation | JAK2V617F n=9 |
| % JAK2V617F VAF at inclusion, median (min-max) | 12,81% (1,36% - 32,58%) |
