# Supplementary figures and images for: An arginase1- and PD-L1-derived peptide-based vaccine for myeloproliferative neoplasms: A first-in-man clinical trial
Source: Front Immunol. 2023 Feb 23;14:1117466. doi: 10.3389/fimmu.2023.1117466 (PMC9996128; doi:10.3389/fimmu.2023.1117466)

## Slide 1
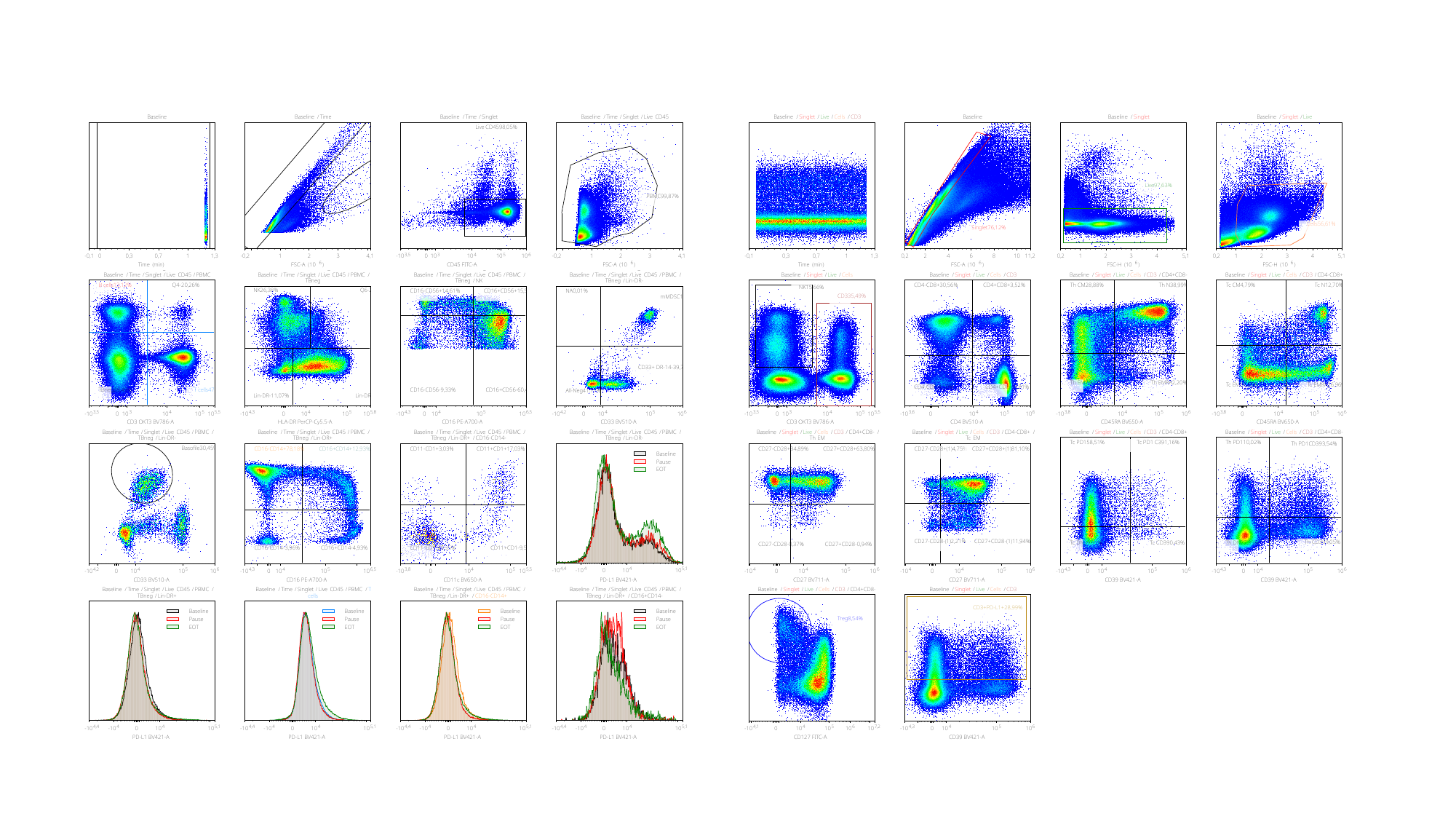

Supplement: Supplementary Figure 2 — Gant chart of the treatment and sample collection schedule. [file Presentation_2.pptx]

## Slide 1
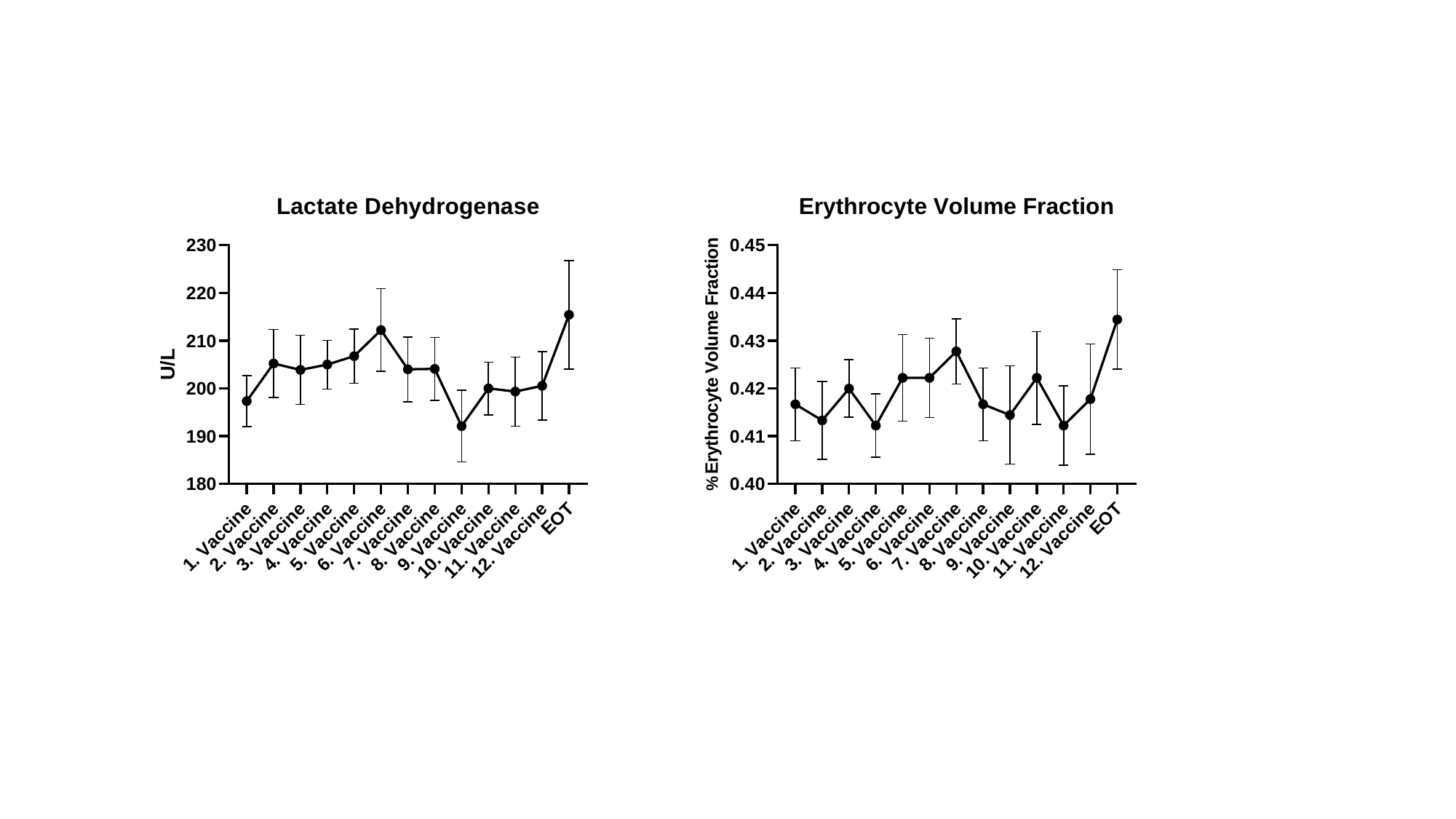

Supplement: Supplementary Figure 3 — Antibodies used in phenotypic PBMC characterization using fluorescence-activated cell sorting. [file Presentation_3.pptx]

## Slide 1
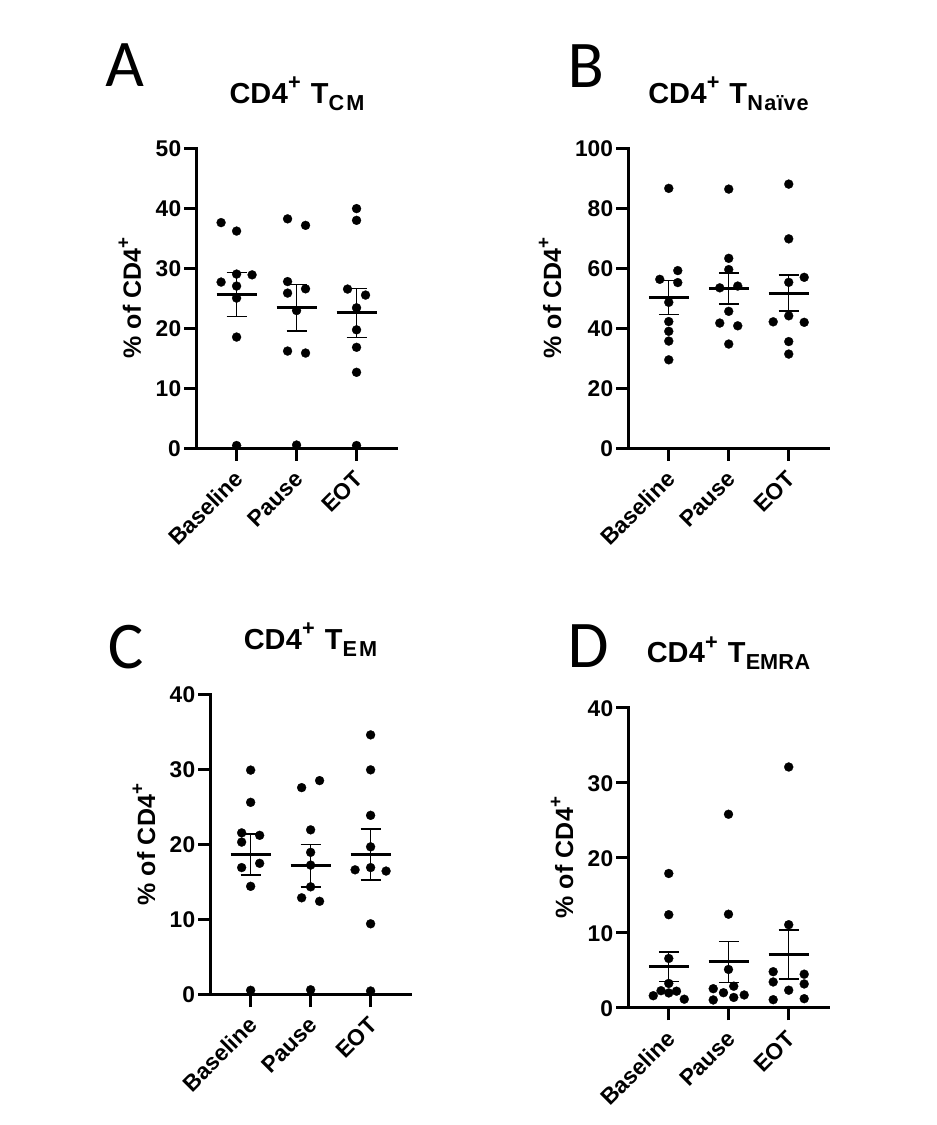

A
B
D
C

Supplement: Supplementary Figure 5 — Analysis of mean lactate hydrogenase (left) and erythrocyte volume fraction (right) in the treated patients (n=9) during the study. Error bars depict the standard error of the mean. [file Presentation_5.pptx]

## Slide 1
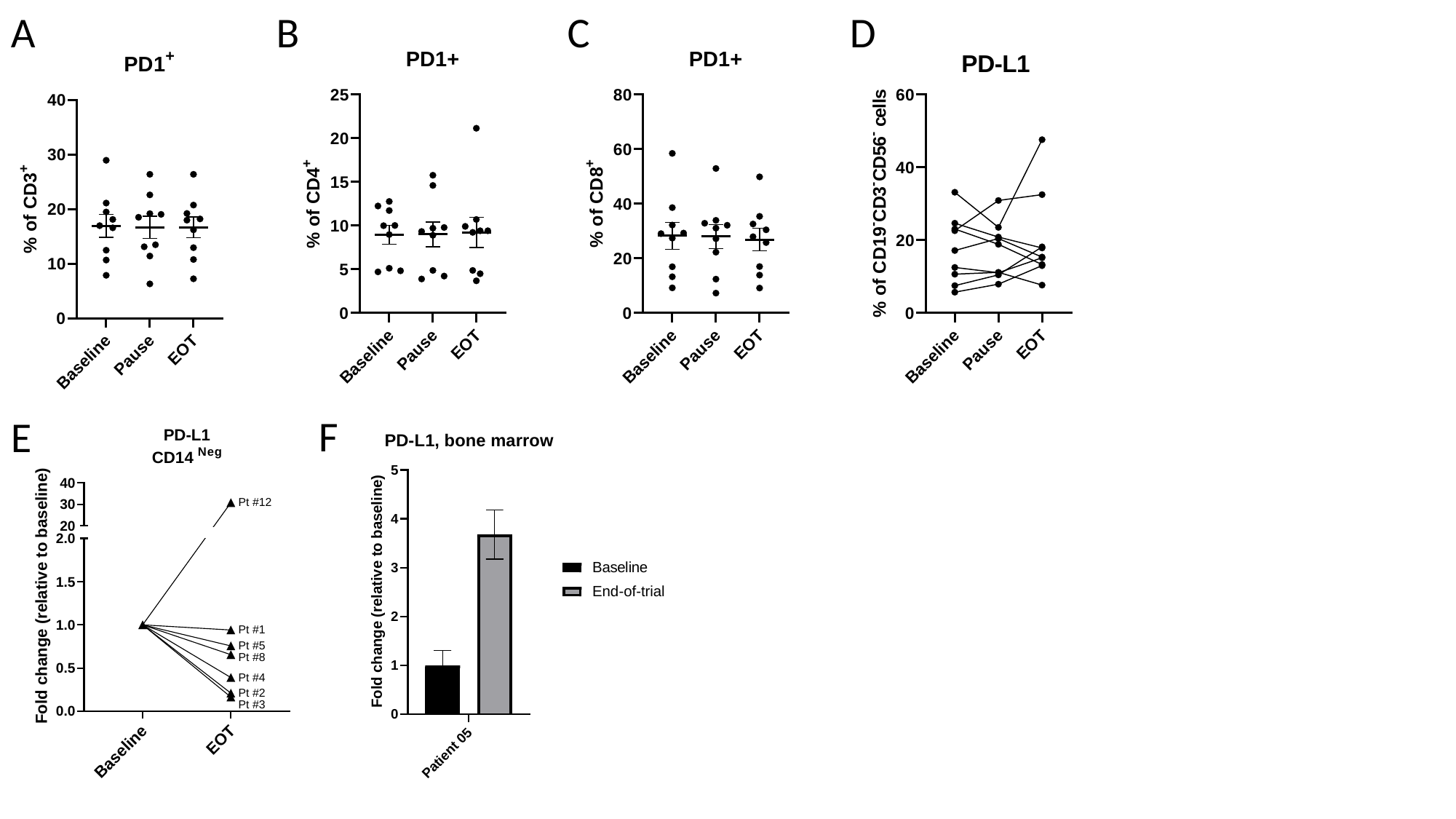

A
B
C
D
F
E

Supplement: Supplementary Figure 6 — Summary of statistical significance analysis of in vitro and ex vivo IFNγ ELISPOT responses to ArgLong2 and PD-L1Long1 during the study shown in and 3. A, B: Statistical analysis summary of in vitro IFNγ ELISPOT responses to ArgLong2 (A) and PD-L1Long1 (B). C. Summary of statistically significant results from ELISPOT assays showing the number of patients with responses to either one epitope or both. D, E: Statistical analysis of ex vivo ELISPOT responses to ArgLong2 (D) and PD-L1Long1 (E). F: Overview of statistically significant results from ELISPOT assays showing the number of patients with responses to one or both vaccine peptides. * indicates statistical significance, based on the DFR method (33); ns-nonsignificant response; DR (for non-triplicate samples only): empirical response defined as true, when at least twice the number of spots were observed in the peptide wells, compared to the number in control wells. [file Presentation_6.pptx]
