# Supplementary material for: An arginase1- and PD-L1-derived peptide-based vaccine for myeloproliferative neoplasms: A first-in-man clinical trial
Source: Front Immunol. 2023 Feb 23;14:1117466. doi: 10.3389/fimmu.2023.1117466 (PMC9996128; doi:10.3389/fimmu.2023.1117466)
Supplement: Supplementary Figure 4 — Fluorescence-activated cell sorting gating strategies, applied with NovoExpress 1.5.1 software. [file Presentation_4.pptx]

## Slide 1
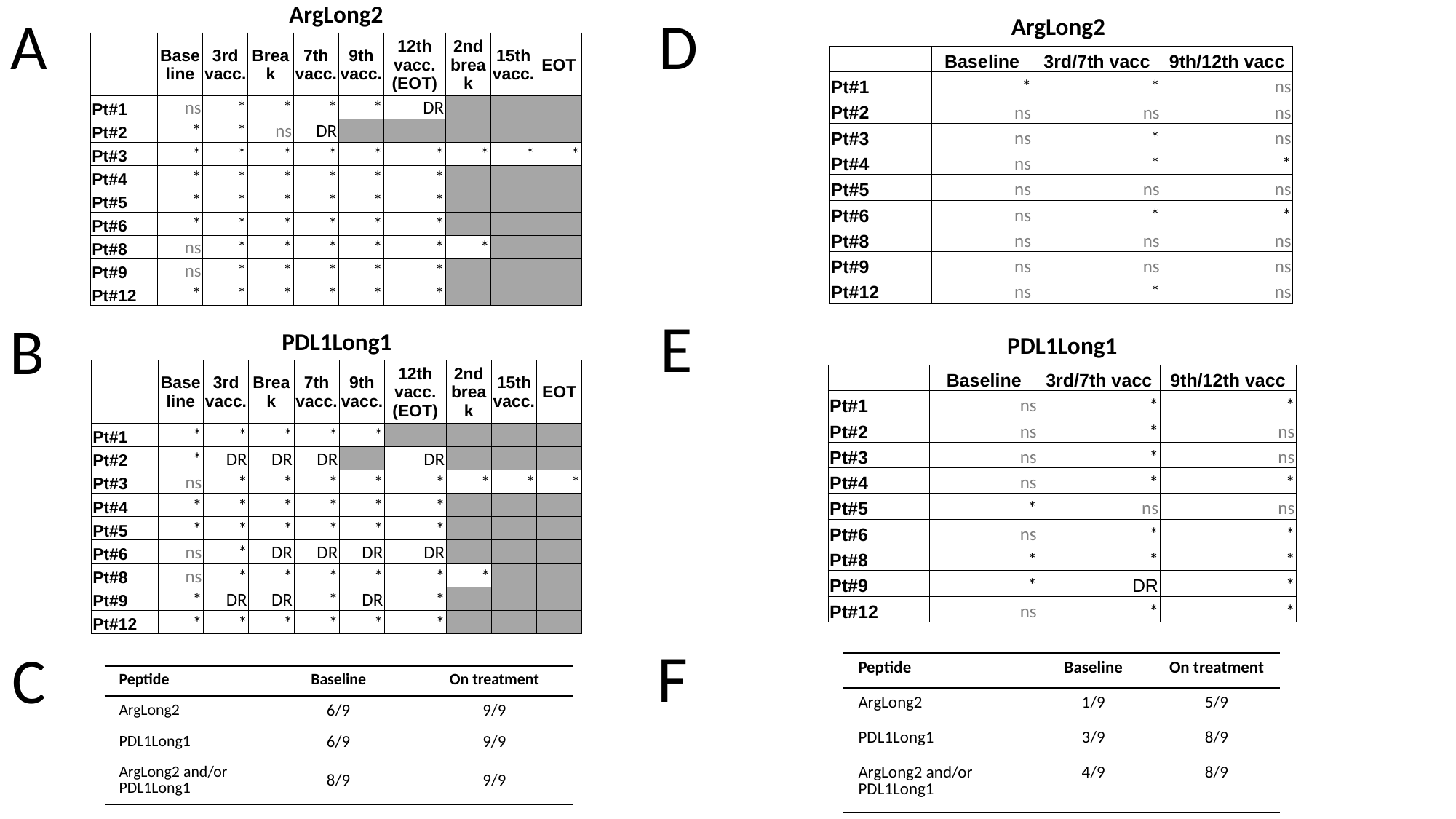

A
| ArgLong2 | | | | | | | | | |
| --- | --- | --- | --- | --- | --- | --- | --- | --- | --- |
| | Baseline | 3rd vacc. | Break | 7th vacc. | 9th vacc. | 12th vacc. (EOT) | 2nd break | 15th vacc. | EOT |
| Pt#1 | ns | \* | \* | \* | \* | DR | | | |
| Pt#2 | \* | \* | ns | DR | | | | | |
| Pt#3 | \* | \* | \* | \* | \* | \* | \* | \* | \* |
| Pt#4 | \* | \* | \* | \* | \* | \* | | | |
| Pt#5 | \* | \* | \* | \* | \* | \* | | | |
| Pt#6 | \* | \* | \* | \* | \* | \* | | | |
| Pt#8 | ns | \* | \* | \* | \* | \* | \* | | |
| Pt#9 | ns | \* | \* | \* | \* | \* | | | |
| Pt#12 | \* | \* | \* | \* | \* | \* | | | |
D
| ArgLong2 | | | |
| --- | --- | --- | --- |
| | Baseline | 3rd/7th vacc | 9th/12th vacc |
| Pt#1 | \* | \* | ns |
| Pt#2 | ns | ns | ns |
| Pt#3 | ns | \* | ns |
| Pt#4 | ns | \* | \* |
| Pt#5 | ns | ns | ns |
| Pt#6 | ns | \* | \* |
| Pt#8 | ns | ns | ns |
| Pt#9 | ns | ns | ns |
| Pt#12 | ns | \* | ns |
E
B
| PDL1Long1 | | | | | | | | | |
| --- | --- | --- | --- | --- | --- | --- | --- | --- | --- |
| | Baseline | 3rd vacc. | Break | 7th vacc. | 9th vacc. | 12th vacc. (EOT) | 2nd break | 15th vacc. | EOT |
| Pt#1 | \* | \* | \* | \* | \* | | | | |
| Pt#2 | \* | DR | DR | DR | | DR | | | |
| Pt#3 | ns | \* | \* | \* | \* | \* | \* | \* | \* |
| Pt#4 | \* | \* | \* | \* | \* | \* | | | |
| Pt#5 | \* | \* | \* | \* | \* | \* | | | |
| Pt#6 | ns | \* | DR | DR | DR | DR | | | |
| Pt#8 | ns | \* | \* | \* | \* | \* | \* | | |
| Pt#9 | \* | DR | DR | \* | DR | \* | | | |
| Pt#12 | \* | \* | \* | \* | \* | \* | | | |
| PDL1Long1 | | | |
| --- | --- | --- | --- |
| | Baseline | 3rd/7th vacc | 9th/12th vacc |
| Pt#1 | ns | \* | \* |
| Pt#2 | ns | \* | ns |
| Pt#3 | ns | \* | ns |
| Pt#4 | ns | \* | \* |
| Pt#5 | \* | ns | ns |
| Pt#6 | ns | \* | \* |
| Pt#8 | \* | \* | \* |
| Pt#9 | \* | DR | \* |
| Pt#12 | ns | \* | \* |
F
C
| Peptide | Baseline | On treatment |
| --- | --- | --- |
| ArgLong2 | 1/9 | 5/9 |
| PDL1Long1 | 3/9 | 8/9 |
| ArgLong2 and/or PDL1Long1 | 4/9 | 8/9 |
| Peptide | Baseline | On treatment |
| --- | --- | --- |
| ArgLong2 | 6/9 | 9/9 |
| PDL1Long1 | 6/9 | 9/9 |
| ArgLong2 and/or PDL1Long1 | 8/9 | 9/9 |
