# Supplementary material for: An arginase1- and PD-L1-derived peptide-based vaccine for myeloproliferative neoplasms: A first-in-man clinical trial
Source: Front Immunol. 2023 Feb 23;14:1117466. doi: 10.3389/fimmu.2023.1117466 (PMC9996128; doi:10.3389/fimmu.2023.1117466)
Supplement: Supplementary Figure 7 — Characterization of CD4+ T-cell subsets in PBMCs of treated patients. A. Fraction of CD4+ central memory (CM) T cells defined as CD3+CD4+CD45RA-CCR7+. B. The fraction of CD4+ naïve T cells defined as CD3+CD4+CD45RA+CCR7+. C. The fraction of CD4+ effector memory (EM) cells defined as CD3+CD4+CD45RA-CCR7-. D. The fraction of CD4+ TEMRA cells defined as CD3+CD4+CD45RA+CCR7-. Statistical analysis was performed using the Wilcoxon signed-rank test. Graphs represent mean values ± standard error of the mean. [file Presentation_7.pptx]

## Slide 1
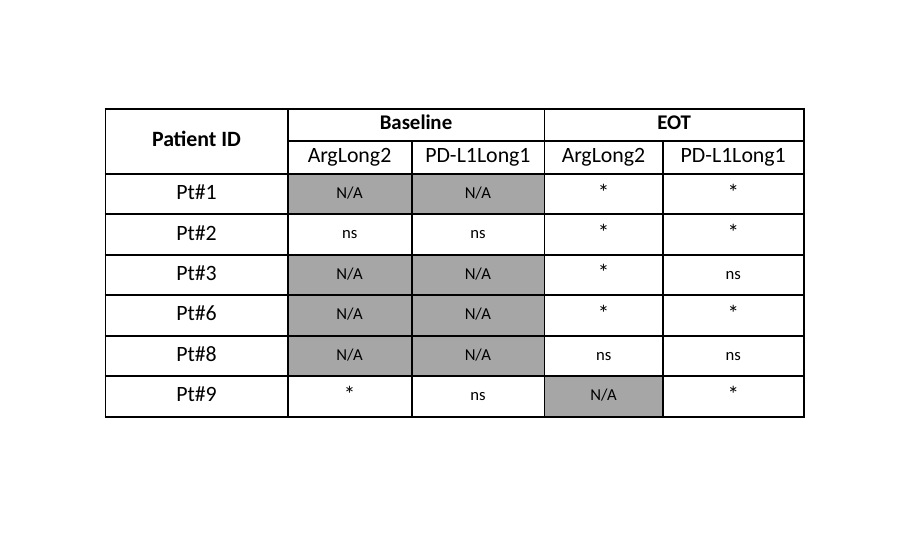

| Patient ID | Baseline | | EOT | |
| --- | --- | --- | --- | --- |
| | ArgLong2 | PD-L1Long1 | ArgLong2 | PD-L1Long1 |
| Pt#1 | N/A | N/A | \* | \* |
| Pt#2 | ns | ns | \* | \* |
| Pt#3 | N/A | N/A | \* | ns |
| Pt#6 | N/A | N/A | \* | \* |
| Pt#8 | N/A | N/A | ns | ns |
| Pt#9 | \* | ns | N/A | \* |
