# Supplementary material for: An arginase1- and PD-L1-derived peptide-based vaccine for myeloproliferative neoplasms: A first-in-man clinical trial
Source: Front Immunol. 2023 Feb 23;14:1117466. doi: 10.3389/fimmu.2023.1117466 (PMC9996128; doi:10.3389/fimmu.2023.1117466)
Supplement: Supplementary Figure 8 — Expression of PD-1 on CD3+ (A), CD4+ (B), CD8+ (C) cells in the peripheral blood as detected by flow cytometric analysis at baseline and during the trial. Graphs represent mean values ± the standard error of the mean. D: FACS analysis of PD-L1 expression on CD19−CD3−CD56− myeloid cells at baseline and during the vaccination trial. E: PD-L1 expression by CD14 negative cells in PBMCs of treated patients before and after treatment as measured by RT-qPCR. F: PD-L1 expression in BMNCs of patient 5 before and after treatment as measured by RT-qPCR. [file DataSheet_1.pdf]

TYPE	Number of patients	Grade 1	Grade 2	Grade 3
**Body odor**	1	1		
**Diarrhea**	1	1		
**Dry skin**	1	1		
**Dysgeusia**	1	1		
**Edema limbs**	1	1		
**Eczema**	1		1	
**Fatique**	3	2	1	
**Flu like symptoms**	3	3		
**Herpes simplex reactivation**	1		1	
**Headache**	1	1		
**Infection**	3	1	2	
**Injection site reaction**	9		9	
**Pain**	2	2		
**Palpitations**	1	1		
**Pruritus**	2	2		
**Rotator cuff injury**	1	1		
**Vasovagal Reaction**	1			1
