# Supplementary material for: An arginase1- and PD-L1-derived peptide-based vaccine for myeloproliferative neoplasms: A first-in-man clinical trial
Source: Front Immunol. 2023 Feb 23;14:1117466. doi: 10.3389/fimmu.2023.1117466 (PMC9996128; doi:10.3389/fimmu.2023.1117466)
Supplement: Supplementary Table 1 — Summary of IFNγ ELISPOT assay results for peptide specific responses in skin infiltrating lymphocytes (SKILS) expanded from delayed type hypersensitivity test in patients with MPN, before and after the trial (EOT). Intradermal injections of the two peptides, dissolved in sterile water and DMSO were biopsied 48 hours after administration. SKILS were expanded in media containing IL2 and harvested. * = significant (DFR) immune response detected; ns = No significant (DFR) immune response detected; N/A – not available due to lack of outgrowth of SKILS. [file DataSheet_2.pdf]

## Inclusion criteria

The patients must fulfill the following criteria:

1. Diagnosis of essential thrombocythemia or Polycythemia Vera, according to the WHO criteria[123,124]
2. Age ≥18 years
3. Performance status ≤ 2 (ECOG-scale)
4. Expected survival > 3 months
5. Sufficient bone marrow function, i.e.
    a. Leucocytes ≥ 1,5 x $10^9$
    b. Granulocytes ≥ 1,0 x $10^9$
    c. Thrombocytes ≥ 20 x $10^9$
    d. Hemoglobin ≥ 5.5 mmol/L
6. Creatinine < 2.5 upper normal limit, i.e. < 300 µmol/l
7. Sufficient liver function, i.e.
    a. ALAT < 2.5 upper normal limit, i.e. ALAT <112 U/l
    b. Bilirubin < 30 U/l
8. For women: Agreement to use contraceptive methods with a failure rate of < 1% per year during the treatment period and for at least 120 days after the last treatment.
9. For men: Agreement to use contraceptive measures and agreement to refrain from donating sperm.

## Exclusion criteria

1. Other malignancies in the medical history excluding basal cell carcinoma. Patients cured for another malignant disease with no sign of relapse five years after ended treatment is allowed to enter the protocol.
2. Significant medical condition per investigators judgement e.g. severe Asthma/COPD, poorly regulated heart condition, insulin dependent diabetes mellitus.
3. Acute or chronic viral or bacterial infection e.g. HIV, hepatitis or tuberculosis
4. Serious known allergies or earlier anaphylactic reactions.
5. Known sensibility to Montanide ISA-51
6. Any active autoimmune diseases e.g. autoimmune neutropenia, thrombocytopenia or hemolytic anemia, systemic lupus erythematosus, scleroderma, myasthenia gravis, autoimmune glomerulonephritis, autoimmune adrenal deficiency, autoimmune thyroiditis etc.
7. Pregnant and breastfeeding women.
8. Fertile women not using secure contraception with a failure rate less than < 1%
9. Patients taking immune suppressive medications incl. systemic corticosteroids and methotrexate at the time of enrollment
10. Psychiatric disorders that per investigator judgment could influence compliance.
11. Treatment with other experimental drugs
12. Treatment with other anti-cancer drugs – except IFN-a, hydroxyurea or anagrelide.
13. Treatment with ruxolitinib.
14. Treatment with chemotherapy or immune therapy (excluding IFN-a, hydroxyurea or anagrelide) within the last 28 days.
