# Supplementary material for: An arginase1- and PD-L1-derived peptide-based vaccine for myeloproliferative neoplasms: A first-in-man clinical trial
Source: Front Immunol. 2023 Feb 23;14:1117466. doi: 10.3389/fimmu.2023.1117466 (PMC9996128; doi:10.3389/fimmu.2023.1117466)
Supplement: Supplementary file 10 [file DataSheet_3.pdf]

	Inclusion	1st round of vaccines						Pause[a]	2nd round of vaccines						End of study
Visit	1	2	3	4	5	6	7	8	9	10	11	12	13	14	15
Week	-4 to 0	0	2	4	6	8	10	4	0	2	4	6	8	10	
Vaccine PD-L1		V	V	V	V	V	V		V	V	V	V	V	V	
Vaccine ARGLong2		V	V	V	V	V	V		V	V	V	V	V	V	
Health blood sample	HB	HB	HB	HB	HB	HB	HB	HB	HB	HB	HB	HB	HB	HB	HB
Study blood sample	SBx2				SB			SB		SB		SB			SB
Doctor visit with CTCAE[b]		CTC	CTC	CTC	CTC	CTC	CTC		CTC	CTC	CTC	CTC	CTC	CTC	CTC
Electrocardiography	ECG							ECG							ECG
Delayed type hypersensitivity	DTH														DTH
Bone marrow aspiration	BM														BM
Spleen status	SS														SS
Next generation sequencing	NGS														NGS

[A]The pause in treatment is estimated to be 6 weeks - but can be prolonged or shortend by the investigator.

[B]doctors visit is reduced after safetyperiod
