# Supplementary material for: An arginase1- and PD-L1-derived peptide-based vaccine for myeloproliferative neoplasms: A first-in-man clinical trial
Source: Front Immunol. 2023 Feb 23;14:1117466. doi: 10.3389/fimmu.2023.1117466 (PMC9996128; doi:10.3389/fimmu.2023.1117466)
Supplement: Supplementary file 11 [file DataSheet_4.pdf]

Panel 1	Distributor	Item name	Item no.
	Miltenyi Biotec	**CD127 (REA614) FITC**	130-113-417
	BD	**HLA-DR (G46-6) PerCP-Cy5.5**	560652
	BD	**CD28 APC**	559770
	BD	**CD8 (RPA-T8) APC-R700**	565165
	Thermo-Fisher	**NIR**	
	BD	**CD39 (TU66) BV421**	563679
	BD	**CD4 (SK3) BV510**	562970
	BD	**CD56 (NCAM16.2) BV605**	562780
	BD	**CD45RA (HI100) BV650**	563963
	BD	**CD27 (M-T271) BV711**	564893
	BD	**CD3 (SK7) BV786**	563800
	Nordic biosite	**CCR7 (G043H7) PE**	353204
	Nordic biosite	**PD-1 (EH12.2H7) PE/Dazzle 594**	329940
	BD	**CD38 PE-CY5**	555461
	AAT BIOQUEST	**CD29 (HI29a) PE/AF700**	102901PI
	BD	**CD25 (2A3) PE-CY7**	335824

Panel 2	Distributor	Varenavn / Item name	Varenr / Item no.
	BD	**CD45 (HI30) FITC**	555482
	Nordic biosite	**CD1c (L161) APC**	331524
	BD	**CD274 PD-L1 (MIH1) BV421**	563738
	BD	**CD33 (WM53) BV510**	563257
	BD	**CD123 (7G3) BV605**	564197
	BD	**CD11c (B-ly6) BV650**	563404
	BD	**CD19 (SJ25C1) BV711**	563036
	BD	**CD34 (R718)**	566973
	BD	**CD14 (MØP9) PE-CFE594**	562335
	Nordic biosite	**CD56 (HCD56) PE-CY7**	318318
